# Experimental demonstration of weak chirality enhancement by hybrid perovskite nanocrystals using photonic spin Hall effect

**DOI:** 10.1515/nanoph-2022-0313

**Published:** 2022-08-10

**Authors:** Zheng Lai, Shuai Lin, Youzhi Shi, Maoxin Li, Guangyou Liu, Bingbing Tian, Yu Chen, Xinxing Zhou

**Affiliations:** International Collaborative Laboratory of 2D Materials for Optoelectronics Science and Technology, Institute of Microscale Optoelectronics, Shenzhen University, 518060, Shenzhen, P. R. China; Key Laboratory of Low-Dimensional Quantum Structures and Quantum Control of Ministry of Education, Synergetic Innovation Center for Quantum Effects and Applications, School of Physics and Electronics, Hunan Normal University, 410081, Changsha, P. R. China

**Keywords:** chiral perovskites, organic–inorganic hybrid perovskite nanocrystals, photonic spin Hall effect, weak chirality

## Abstract

Chiral perovskites have attracted considerable attention as excellent spin-emitting materials for applications in spintronics, quantum optics, and biological. Especially in drug development of biological, weak chirality molecules are frequently selected to reduce the side effects of toxics, and there is a common defect for accurately detecting the weak chirality with common methods at room temperature. In this study, formamidine lead bromide perovskite nanocrystals (FAPbBr_3_ NCs) were coated with chiral ligands, whose chirality was too weak to be observed in the visible region at room temperature. Thus, by characterizing the transverse shift of photonic spin Hall effect (SHE), the accurate discrimination of weak chirality in the visible region was achieved successfully. By measuring the shift value and light spot splitting of photonic SHE at the same concentration, NEA-coated FAPbBr_3_ NCs can effectively enhance the chirality of naphthalene ethylamine (NEA) ligands when under the mutually reinforcement of chiral molecular and inorganic parts. In addition, we furtherly clearly distinguished the tiny chiral distinction of NEA-coated FAPbBr_3_ NCs with different particle sizes, which revealed that the chirality decreases with the increase of particle size. These findings could provide effective solutions for the detection and application of weak chirality in hybrid perovskite nanocrystals in universal environment.

## 1 Introduction

Chirality occurs widely in nature and is an important symmetry characteristic in several disciplines. Since the chiral properties have been successfully introduced into optics, nanostructures, and nano-systems [1], chiral perovskites play a vital role because of their excellent properties and have been applied in several fields, such as spintronics, circularly polarized light detectors, and quantum optics [2], [3], [4]. Recently, benefitting from its important role in biological applications of weak chirality [5, 6], studies on chiral perovskites have made some significant progress [7, 8]. Coating nanocrystals with chiral ligands is an important method for perovskites acquiring chirality [9], [10], [11]. However, the weak chirality of perovskites is difficult to detect with common methods at room temperature [12], [13], [14], [15], [16]. Hence, it is imperative to develop an efficient and accurate strategy for weak chirality measurements at more universal environment and then to in-depth understand the interaction of chiral molecules with nanocrystals such as particle size [17], [18], [19]. Considering its high sensitivity to some physical parameters, universality of environment width, and flexibility of operating wavelength [20], [21], [22], [23], the photonic SHE combined with a weak measurement system has attracted significant attention in the field of precision measurement, observation of photon trajectory, sensor, and spin-sensitive devices [24], [25], [26], [27], [28], [29], [30]. Noting that the optical rotation (OR) angle caused by chirality is sensitive to polarization state of light in photonic SHE [31], [32], [33], [34], therefore, combination of photonic SHE with a weak measurement system can be a promising option to reveal the weak chirality of perovskites.

In this study, a weak measurement system for the photonic SHE was established in the visible region to identify the weak chirality of perovskites at room temperature. In order to demonstrate the chirality detection via photonic SHE, the S-/R-/rac-NEA–coated FAPbBr_3_ NCs were prepared for comparison (S and R (Sinister and Rectus) have equal and opposite OR angle, while rac (racemate) does not exhibit any OR angle). Chirality enhancement of FAPbBr_3_ NCs coated with chiral ligands can be confirmed by comparing the OR angle and light spot splitting of non–NEA-coated FAPbBr_3_ NCs, NEA ligands, and NEA-coated FAPbBr_3_ NCs. In addition, the chirality gradually decreased with an increase of the particle size of NEA-coated FAPbBr_3_ NCs. These results compensate for the difficulty of identifying weak chirality at room temperature and prove the application value of photonic SHE as a simple and efficient method to identify weak chiral perovskites.

## 2 Results and discussion

**Figure 1:**
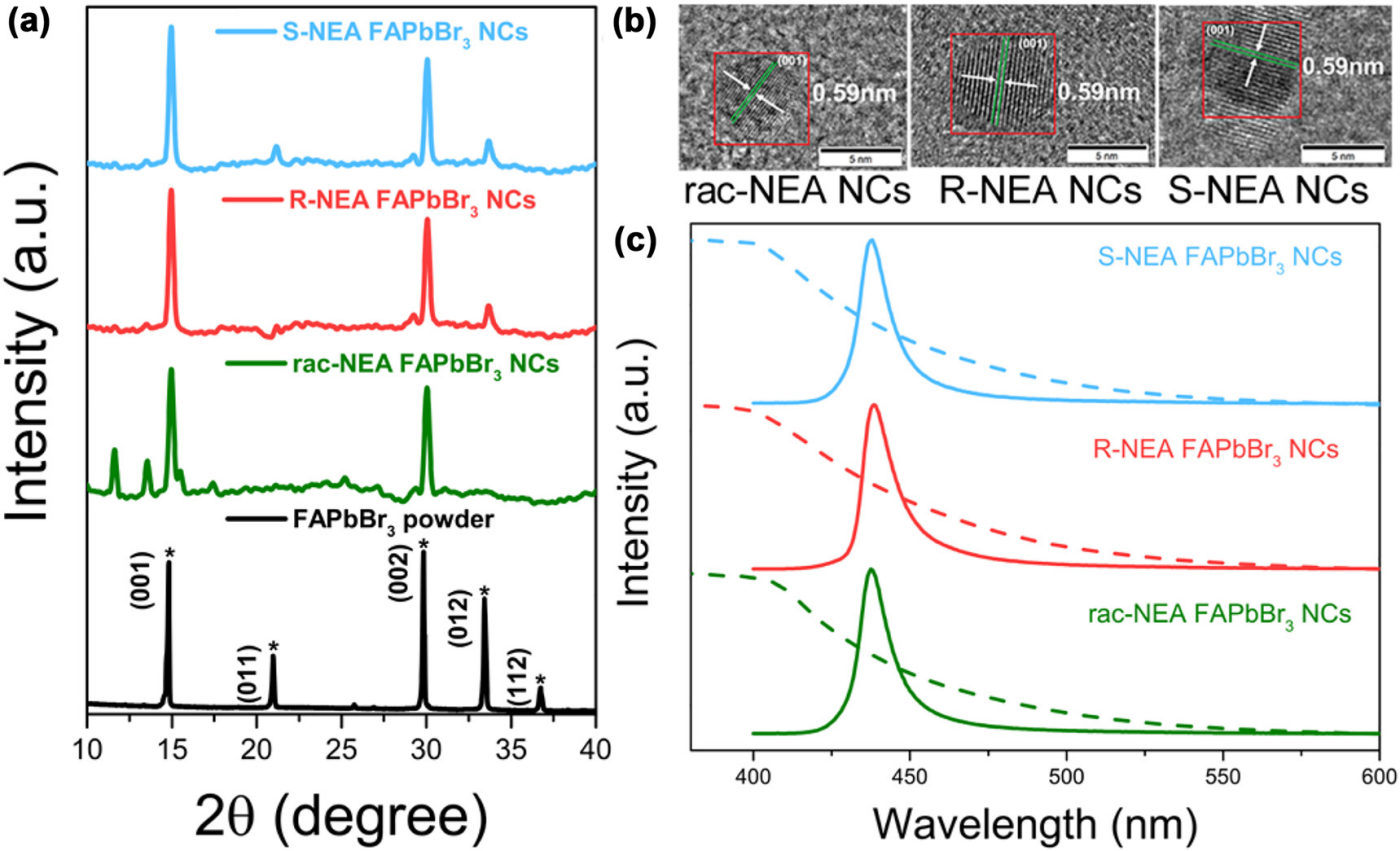
Characterization of the morphology and properties of S/R/rac-NEA-coated FAPbBr3 NCs. (a) XRD spectra and (b) TEM micrographs of FAPbBr_3_ NCs coated with different NEA ligands; (c) absorption spectra (dotted lines) and photoluminescence spectra (solid lines, excitation wavelength of 365 nm) of FAPbBr_3_ NCs coated with different NEA ligands.

First, S-/R-/rac-NEA–coated FAPbBr_3_ NCs were prepared by ligand-assisted reprecipitation (LARP, see Method 1 in the Supplementary Material) [35], [36], [37]. The X-ray diffraction (XRD) patterns of NEA-coated FAPbBr_3_ NCs are shown in Figure 1a, which exhibit a typical cubic crystal formed with a lattice spacing of 0.59 nm on the (001) plane, respectively. The NEA-coated FAPbBr_3_ NCs have a typical three-dimensional crystal structure. The S-/R-/rac-NEA–coated FAPbBr_3_ NCS are analyzed using HR-TEM shown in Figure 1b. The NEA-coated FAPbBr_3_ NCs all have similar particle size, along with typical (001) lattice planes a 0.59 nm interplanar spacing. The HR-TEM images of non–NEA-coated FAPbBr_3_ NCs with the same particle size are shown in Figure S1. The absorption spectra and photoluminescence spectra of three different chiral NEA-coated FAPbBr_3_ NCs are shown in Figure 1c, with the typical narrowband emission of perovskite nanocrystals. To distinguish the chirality of these NCs, three types of NEA-coated FAPbBr_3_ NCs were completely dispersed in hexane colloids at identical concentrations (0.1 mg/mL) (see Method 1 in the Supplementary Material for details). The circular dichroism (CD) spectra at 300–800 nm is shown in Figure S2, where the chirality of three NEA-coated FAPbBr_3_ NCs all cannot be distinguished. The chirality of S-/R-NEA ligands had been investigated previously and exhibited opposite signals at 320 nm, whereas rac-NEA ligands had no CD absorption peak [38].

**Figure 2:**
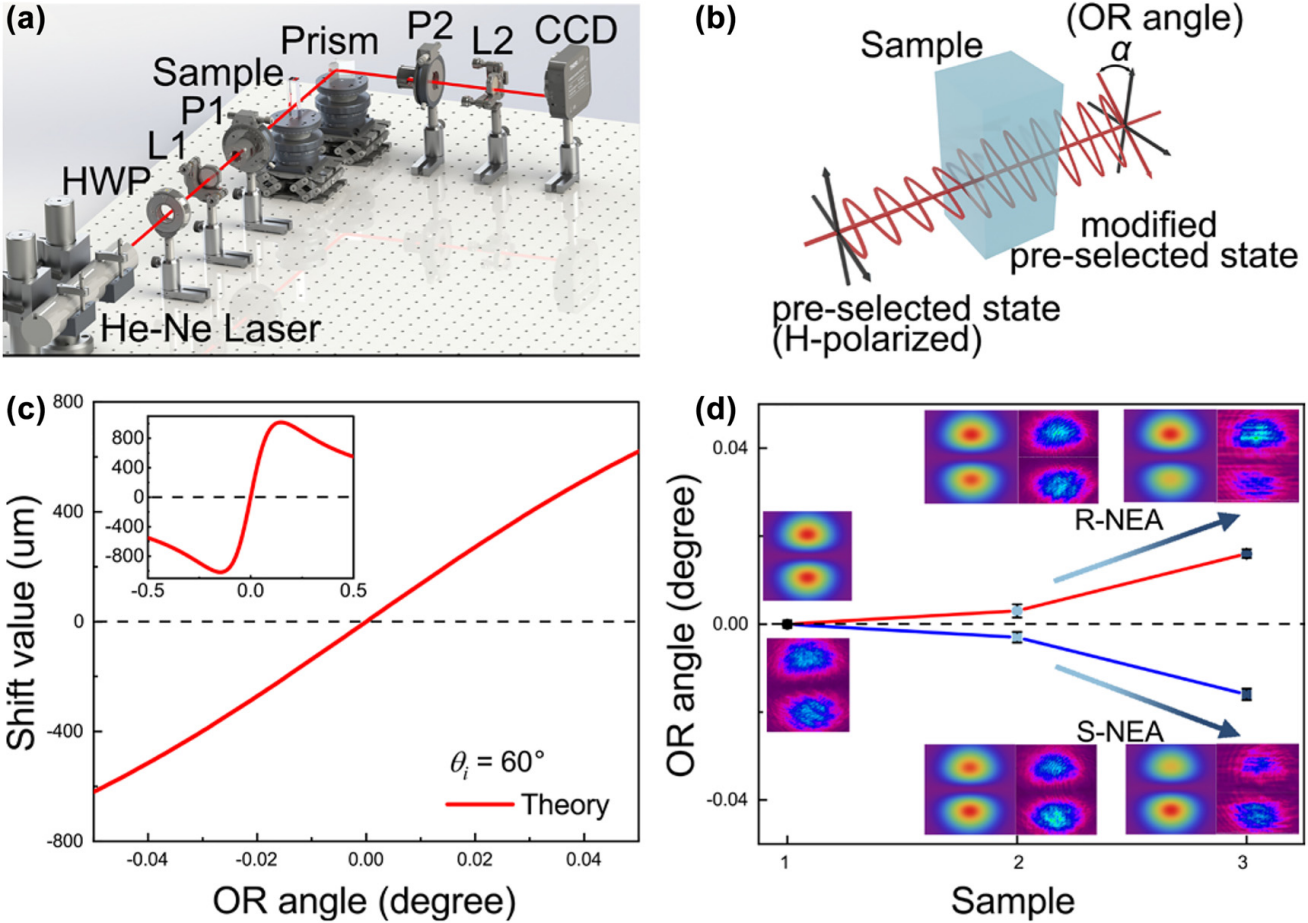
The experimental demonstration in photonic SHE. (a) Experimental scheme for the weak measurement system of photonic SHE; (b) schematic diagram of OR angle provided by chirality concerning the plane of polarization of incident light; (c) theoretical relationship between OR angle and amplified transverse shift of photonic SHE in the weak measurement system; (d) the observation results of OR angle of sample (1: non–NEA-coated FAPbBr_3_ NCs, 2: NEA ligands, and 3: NEA-coated FAPbBr_3_ NCs) derived by amplified transverse shift; the insert shows light spot splitting of the photonic SHE in theory and experiment.

In particularly, it is considered that the value of OR angle directly represents the chirality [21, 25, 33]. Hence, to distinguish the chirality of NEA-coated FAPbBr_3_ NCs and NEA ligands, we designed a weak measurement system for photonic SHE in the visible region (*λ* = 632 nm) to detect the OR angle, as shown in Figure 2a (see Methods 3 in the Supplementary Material for details on theoretical calculation). And Figure 2b reveals the influence of OR angle provided by chirality on the polarization of incident light when light passes through the sample.

During the experiment, the incident angle was fixed at 60°, approaching the Brewster angle for a large amplifiedtransverse shift of the photonic SHE. We first demonstrate the theoretical relationship between the transverse shift and the OR angle. The relationship on a large scale (−0.5 to 0.5°) is displayed in the inset of Figure 2c. Most importantly, for weak chirality, we focused on the small scale of the OR angle, as shown by the solid red line in Figure 2c. Under the completely orthogonal between preselected state (H) and postselected state (V), the OR angle caused by chiral molecules was introduced as only one variate into the preselected sate. This tiny OR angle that changes the polarization state of incident light will obviously affect the amplified transverse shift of photonic SHE. The theoretical relationship between OR angle and shift value based on Eq. S(8) is shown in Figure 2c (see Methods 3 in the Supplementary Material for details on theoretical calculation), which reveals an approximately linear relation. Therefore, the corresponding OR angle can be deduced by bringing the shift value collected from a charge-coupled device (CCD) into the curve in Figure 2c based on Eq. S(8).

We then investigated the weak chirality of non–NEA-coated FAPbBr_3_ NCs, NEA ligands, and NEA-coated FAPbBr_3_ NCs at the same concentration (0.1 mg/mL) for comparison. After measuring the transverse shift of photonic SHE, the corresponding OR angle is shown in Figure 2d. It can be clearly seen that the amplified transverse shift of non–NEA-coated FAPbBr_3_ is 0, which reveals the FAPbBr_3_ NCs itself provides no OR angle. The OR angle of the NEA-coated FAPbBr_3_ NCs (0.016° ± 0.0013°) was significantly larger than that of the NEA ligands (0.003° ± 0.0012°). With an increase in the OR angle of NEA ligands and NEA-coated FAPbBr_3_ NCs, the light intensity distribution gradually evolved from the complete symmetric distribution to asymmetric splitting. Comparing the light spot in inset of Figure 2d, it is evident that the spot splitting of NEA-coated FAPbBr_3_ NCs under the same chiral direction is clearer. Simultaneously, the opposite chirality of providing the opposite OR angle will lead to an equal shift value and opposite splitting direction, which can be further illustrated by the evolution of light spot splitting. The experimental data of light spot are consistent with the theory. Therefore, it can be inferred that chirality can be effectively enhanced by NEA-coated FAPbBr_3_ NCs at the amplified transverse shift in photonic SHE is accurate and same concentration.

Mutual reinforcement causes chiral molecules to deliver their chirality to the inorganic part, whereas the structural relaxation of the inorganic part influences the organic cation [39, 40]. Moreover, it also proves that the reliable in detecting weak chirality.

**Figure 3:**
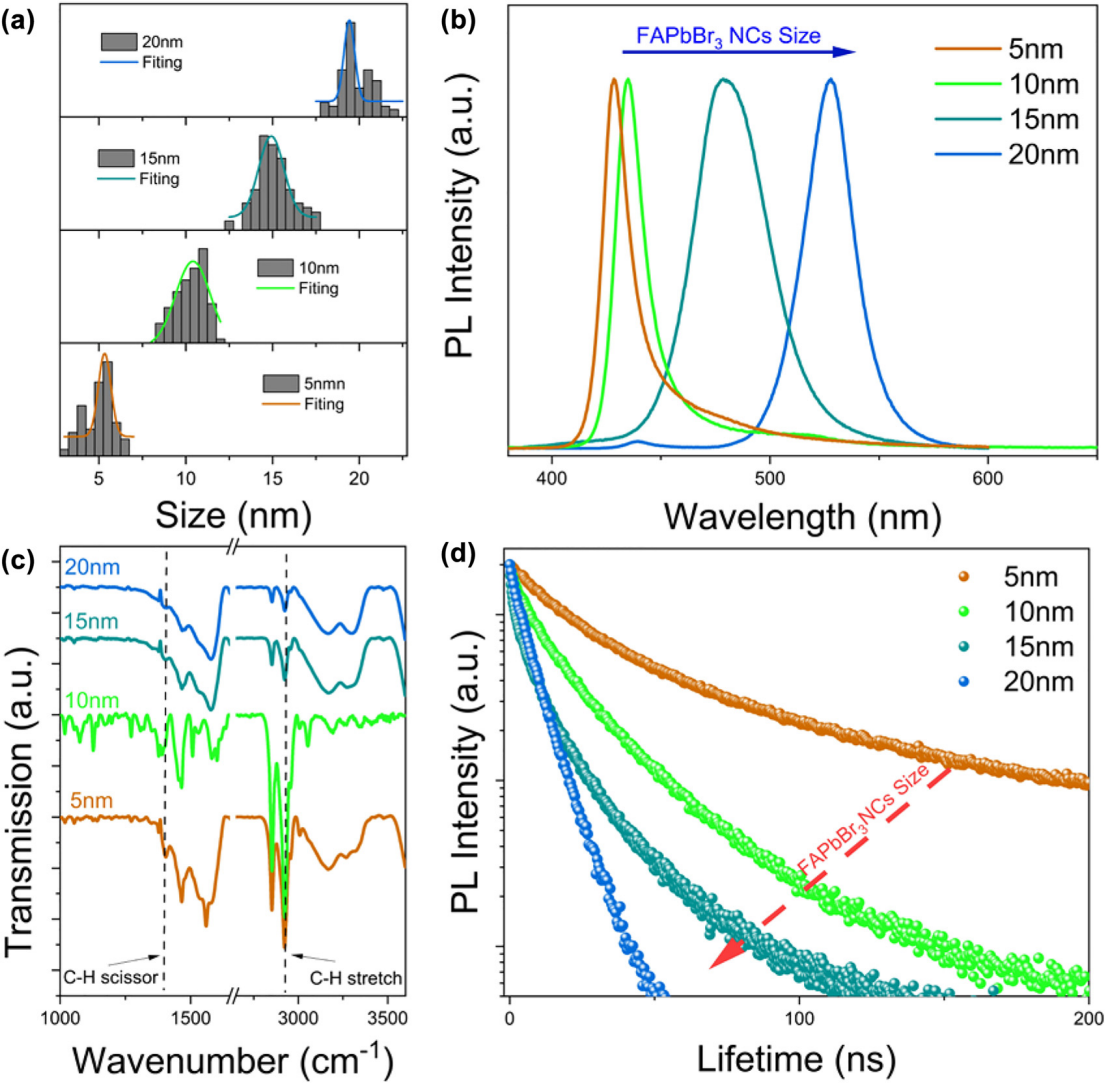
Characterization of NEA coated FAPbBr3 NCs with different particle sizes. (a) Particle size distribution histogram of four NEA-coated FAPbBr_3_ NCs; (b) photoluminescence spectra of NEA-coated FAPbBr_3_ NCs with different particle sizes; (c) FT-IR spectra of four NEA-coated FAPbBr_3_ NCs showing the presence of the characteristic bond of the NEA ligands; (d) time-resolved photoluminescence spectra of FAPbBr_3_ NCs with four particle sizes.

To investigate the effect of particle size on the chiral optics of NEA-coated FAPbBr_3_ NCs, FAPbBr_3_ NCs with different particle sizes were prepared (see Method 2 in the Supplementary Material for further details). The particle sizes are shown in Figure 3a and distributed into four sizes. The NEA-coated FAPbBr_3_ NCs exhibit cubic shapes, as shown in the TEM images in Figure S5. We performed photoluminescence (PL) for broad-spectra tunable luminescence, as shown in Figure 3b. As the size of the NCs changed from 5 to 10 nm, the emission wavelength could be adjusted from 426 to 528 nm. The luminescence of NEA-coated FAPbBr_3_ NCs with different particle sizes in UV light under dark conditions is shown in Figure S6. To understand the relationship of the interaction between NEA and particle size of FAPbBr_3_ NCs, Fourier transform infrared spectroscopy (FT-IR) measurements were performed, as shown in Figure 3c. With a decrease of particle size, the intensity of the bending vibration of the C–H bond (1465 cm^−1^) increased slightly, and the stretching vibration of the C–H bond gradually became clearer at 2850–3100 cm^−1^. It revealed that the interaction between NEA^+^ and NCs became gradually stronger with a decrease of particle size [41, 42]. When the particle size of FAPbBr_3_ NCs decreased, the specific surface area increased, and the NEA coated on the surface of single FAPbBr_3_ NCs increased, which may be the reason why the chirality of NEA-coated FAPbBr_3_ NCs increased with a decrease of particle size. In Figure S7, the diffraction (001) peak of the XRD pattern gradually blue-shift with an increase of NC particle size. In Figure S7, the size of the nanocrystal is small with low crystallinity, leading to a low crystal phase content; therefore, the peak intensity of the XRD pattern is weak [43]. The time-resolved PL (TRPL) spectra under different particle sizes are shown in Figure 3d. The lifetime decreased from 16 to 5 ns with an increase in particle size. The shortened lifetime indicates the increased nonradiative recombination of FAPbBr_3_ NCs, which may be related to the reduction of NEA ligands and increase of defects on the surface of FAPbBr_3_ NCs [44]. The same TRPL test was performed on the non–NEA-coated FAPbBr_3_ NCs (Figure S8), and it was found that the lifetime of non–NEA-coated FAPbBr_3_ NCs increased with the increase of particle size due to the exciton confinement at this time, the exciton binding energy increases [45]. This indicates that the interaction between NEA ligands and FAPbBr_3_ NCs stronger as the particle size of NCs decreases. This may be related to the increased specific surface area of FAPbBr_3_ NCs [46]. In Figures S9–S12, we obtained the PL spectra of NEA-coated FAPbBr_3_ NCs and non–NEA-coated FAPbBr_3_ NCs versus time for different particle sizes for comparison. The intensity attenuation rate of the NEA-coated FAPbBr_3_ NCs PL was lower. The emission peaks of the NCs were red-shifted with an increase in particle size, which proves that the addition of NEA improves the stability of the nanocrystals because NEA reduces the nonradiative recombination of FAPbBr_3_ NCs [47].

**Figure 4:**
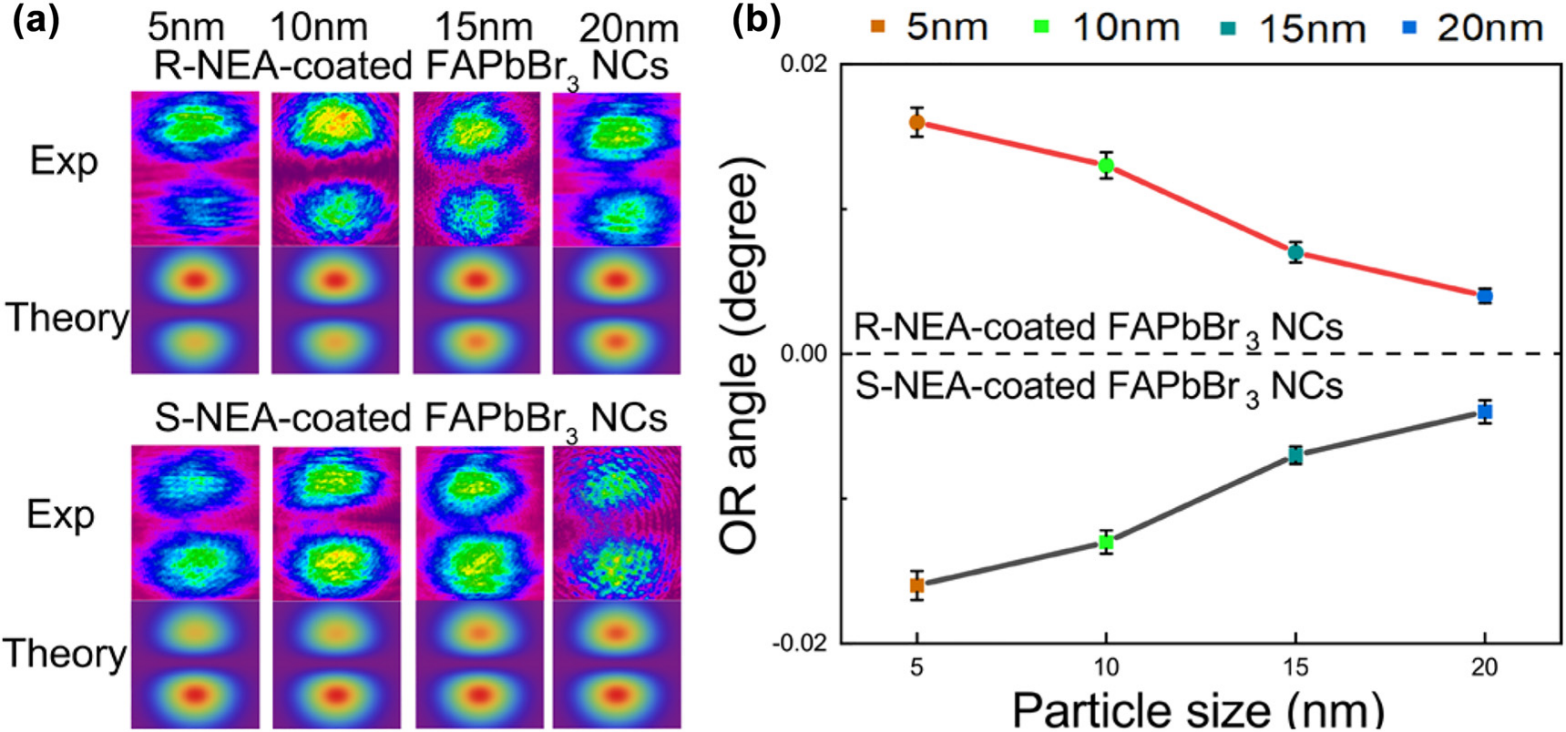
The chirality results of NEA-coated FAPbBr3 NCs with different particle sizes in photonic SHE.(a) The variation of the light spot splitting in theory and experiment, and (b) the OR angle with NEA-coated FAPbBr_3_ NCs particle sizes of 5–20 nm.

Next, we further investigated the influence of the particle size of NEA-coated FAPbBr_3_ NCs on chirality at the same concentration, as shown in Figure 4. Figure 4a shows that the light spot splitting under different particle sizes gradually weakened with an increase in particle size, indicating that the centroid of the beam distribution gradually moved down. The relationship between the OR angle and NEA-coated FAPbBr_3_ NC particle size is shown in Figure 4b. The corresponding OR angle gradually decreased from 0.016° to 0.004° as the particle size increased from 5 to 20 nm with a nearly linear evolution process. In addition, even when the particle size of NEA-coated FAPbBr_3_ NCs was 20 nm, the OR angle was still larger than that of the NEA ligands. Therefore, the chirality of chiral perovskites can be controlled by varying the particle size at the same concentration. This proves that the chirality of NEA-coated FAPbBr_3_ NCs increases with the decrease of particle size, which also indicates that the interaction between NEA ligands and FAPbBr_3_ NCs is stronger. This is consistent with the results of FI-TR and TRPL. Furthermore, we propose a viable alternative for distinguishing particle size with a lower cost and simpler system.

## 3 Conclusions

In summary, NEA-coated FAPbBr_3_ NCs were successfully prepared, and the effectiveness of the synthesis was verified by HR-TEM and XRD analyses. For compensating the invalid chiral characterization by traditional CD spectra at room temperature, the photonic SHE combined with a weak measurement system was utilized to effectively distinguish the weak chirality of NEA ligands and NEA-coated FAPbBr_3_ NCs in the visible region. Results show that NEA-coated FAPbBr_3_ NCs can enhance the chirality of NEA ligands at the same concentration when FAPbBr_3_ NCs itself provides no chirality. In addition, by adjusting the particle size of NEA-coated FAPbBr_3_ NCs using a similar synthesis method, it was found that the interaction between NEA^+^ and NCs is stronger with decrease of particle size. Besides, the chirality gradually increased with the decrease of particle size. These results highlight the potential of photonic SHE in identifying weak chiral materials and promote weak chirality research in hybrid perovskite nanocrystals.

## Supplementary Material

Supplementary Material Details
