# Supplementary material for: Experimental demonstration of weak chirality enhancement by hybrid perovskite nanocrystals using photonic spin Hall effect
Source: Nanophotonics. 2022 Aug 10;11(18):4245–51. doi: 10.1515/nanoph-2022-0313 (PMC11614339; doi:10.1515/nanoph-2022-0313)
Supplement: Supplementary file 1 — Supplementary Material Details [file j_nanoph-2022-0313_suppl.pdf]

supplementary material for

Experimental demonstration of weak chirality enhancement by hybrid perovskite nanocrystals using photonic spin Hall effect

Zheng Lai[1, #], Shuai Lin[1, #], Youzhi Shi[1], Maoxin Li[1], Guangyou Liu[1], Bingbing Tian[1], Yu Chen[1, *], and Xinxing Zhou[2, *]

[1]*International Collaborative Laboratory of 2D Materials for Optoelectronics Science and Technology, Institute of Microscale Optoelectronics, Shenzhen University, Shenzhen 518060, P.R. China*

[2]*Key Laboratory of Low-Dimensional Quantum Structures and Quantum Control of Ministry of Education, Synergetic Innovation Center for Quantum Effects and Applications, School of Physics and Electronics, Hunan Normal University, Changsha 410081, P. R. China*

[#] Zheng Lai and Shuai Lin equally contributed to this work

*Corresponding authors: yuchen@szu.edu.cn, xinxingzhou@hunnu.edu.cn

Chemicals

All reagents were used as received and not further purified. Lead bromide ($PbBr_2$, 99%), Formamidinium acetate (FA-acetate, 99%), oleic acid (OA, technical grade 90%), oleylamine (OAm, technical grade 70%), 1-octadecene (ODE, technical grade 90%), hexane (reagent grade ≥95%), were purchased from Shanghai Macklin Biochemical Corp. (S)-(−)-1-(2-Naphthyl) ethylamine (≥98%) and (R)-(+)-1-(2-naphthyl) ethylamine (≥98%) were purchased from Tokyo Chemical Industry, and 1-(2-naphthyl) ethylamine (≥98%) was purchased from MERYER.

method 1 Synthesis of $FAPbBr_3$ NCs coated with NEA with different chirality.

0.74 mmol of PbBr2 and 25 mL of ODE were added to the three-necked flask, and argon was pumped 3 times at 120 °C and stirred for 0.5 h. Then 4mL of OA and 2mLOAm was injected into the bottle, and then argon was pumped 3 times, heated, and stirred for 1h, so that $PbBr_2$ was completely dissolved. In another three-necked flask was added with 15mmol of FA acetate, 15mL of LODE, and 15mL of OA, and heated and stirred for 1h at 120°C under argon for 3 times. The $PbBr_2$-ODE mixture was cooled to 60 °C, 5 mL of FA-OA mixture was rapidly injected, and then quickly quenched in ice water. The quenched mixed solution was centrifuged at 7500 rpm for 5 min, the obtained precipitate was dispersed in 5 mL of n-hexane, 20 mL of ethyl acetate was added, the precipitate reappeared, and then centrifuged at 7500 rpm for 5 min; this operation was repeated three times, and then vacuum dried. 10 mg of $FAPbBr_3$ NCs were dispersed in 5 mL of n-hexane, 5 mL of NEA n-hexane precursor (5 mg/mL) solution was added, and sonicated for 5 min to obtain NEA-coated $FAPbBr_3$ NCs dispersion.

method 2 Synthesis of NEA-coated $FAPbBr_3$ NCs with different particle sizes.

0.74 mmol of PbBr2 and 25 mL of ODE were added to the three-necked flask, and argon was pumped 3 times at 120 °C and stirred for 0.5 h. Then 4mL of OA and 2mLOAm was injected into the bottle, and then argon was pumped 3 times, heated, and stirred for 1h, so that $PbBr_2$ was completely dissolved. In another three-necked flask was added 15mmol of FA acetate, 15mL of LODE, and 15mL of OA, and heated and stirred for 1h at 120°C under argon for 3 times. The $PbBr_2$-ODE was cooled to 60, 80, 100, and without cooling injected into 5 mL of FA-OA mixture, respectively. $FAPbBr_3$ NCs with different particle sizes were obtained. The remaining steps are the same as in Method 1.

Characterization of NEA-coated $FAPbBr_3$ NCs

The crystal structures of $FAPbBr_3$ NCs were recorded by X-ray diffraction (XRD) (Ultima IV, Rigaku) at 40 kV and 40 mA (Cu Kα X-ray radiation source) with scanning speed and step interval of 4°/min and 0.02°. Fourier transform infrared spectroscopy (FT-IR) (VERTEX 70 V IR spectrometer, Bruker) was measured in the spectral region from 400 to 4000 cm^{-1} with KBr as a beam splitter. The UV-visible absorption spectra was measured by UV–visible spectrophotometer (Cary300, Agilent) ranging from 380 to 600 nm. The PL emission and time-resolved fluorescence decay curves of the $FAPbBr_3$ NCs were performed by a spectrofluorometer (FS5, Edinburgh). CD measurements and UV–visible optical absorption spectra were obtained with a JASCO950 CD spectrometer over a range of 350–600 nm. Transmission electron microscopy images were collected using a JEOL-F200 microscope operated at 200 kV with a beam current of less than 1 pA, applying a low-dose acquisition strategy to avoid beam damage during the acquisition.

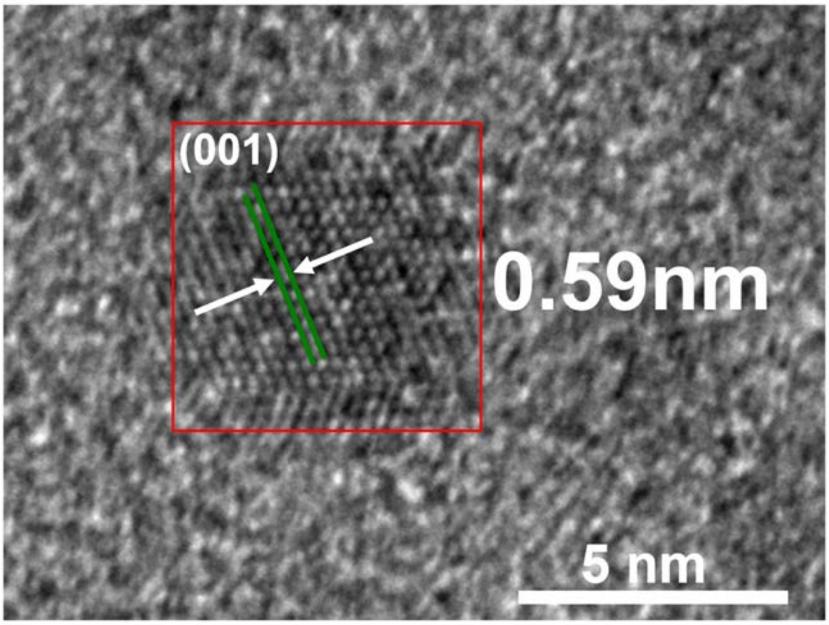

Figure S1. $FAPbBr_3$ NCs synthesized without NEA coating.

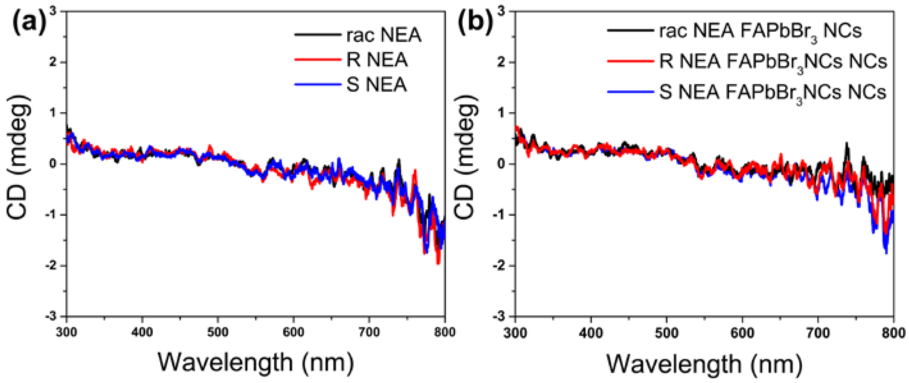

Figure S2. CD spectra of (a) rac-, R- and S-NEA ligands and (b) rac-, R- and S-NEA-coated $FAPbBr_3$ NCs.

Method 3 The calculation method for detecting the optical rotation angle of materials by amplified transverse shift of photonic SHE based on a weak measurement system.

The experimental scheme for the weak measurement system is shown in Figure 2(a). The linearly polarized Gaussian light beam at 632.8 nm is generated by a He-Ne laser, then followed by a half-wave plate (HWP) to fine control the light beam intensity. Next, the Gaussian light beam passes through a short-focus lens (f = 50mm) and the first Glan polarizer (P1), by which the horizontally (H) polarized light beam can be produced as the initial pre-selected state. The post-selected state is kept as vertically polarization which satisfying the pre-selected and post-selected states remain completely orthogonal. The light beam reflects from the surface of the BK7 prism, which is mounted on a rotatable stage to adjust the incident angle as 60°. After that, a long-focus lens (f = 250mm) is used for light beam collimation. These two lenses form an optical confocal cavity, which introduces a propagation amplification

mechanism. Combining the weak value amplification and propagation amplification mechanism, the left and right circularly polarized (LCP and RCP) light beam with the opposite spin splitting caused by disruptive interference can be collected by CCD. When there is no sample in the weak measurement system, the amplified transverse shift is 0 with strictly symmetrical light spot, indicating the OR angle is also 0.

Then, the chiral solution loaded into the sample container is placed in the pre-selected state of a weak measurement system. The tiny OR angle provided by the chirality will change the polarization state of incident light, which will affect the amount of shift. Therefore, the change of shift value is only caused by chiral molecules, and finally the corresponding tiny OR angle can be calculated by large shift value.

The reflection coefficient at the air-glass interface can be derived as:

$$r_p=\frac{n^2\cos\theta_i-\sqrt{n^2-\sin^2\theta_i}}{n^2\cos\theta_i+\sqrt{n^2-\sin^2\theta_i}}, \tag{S1a}$$

$$r_s=\frac{\cos\theta_i-\sqrt{n^2-\sin^2\theta_i}}{\cos\theta_i+\sqrt{n^2-\sin^2\theta_i}}, \tag{S1b}$$

r_p and r_s represent the Fresnel reflection coefficients for parallel and perpendicular polarizations, respectively. θ_i denotes the incident angle and n = 1.515 is the refractive index of the BK7 prism.

Here, we consider the waist of incident light as 24.76 um. The angular spectrum of incident Gaussian light beam can be assumed as:

$$\widetilde{E}_i(k_{ix}+k_{iy})=\frac{w_0}{\sqrt{2\pi}}\exp\left[-\frac{w_0{}^2(k_{ix}{}^2+k_{iy}{}^2)}{4}\right]. \tag{S2}$$

Through the coordinate rotation, the reflected angular spectrum can be calculated [1, 2]:

$$\begin{bmatrix}\widetilde{E}_r^{\mathrm{H}}\\ \widetilde{E}_r^{\mathrm{V}}\end{bmatrix}=\begin{bmatrix} r_p & \frac{k_{ry}\cot\theta_i(r_\mathrm{p}+r_\mathrm{s})}{k_0}\\ -\frac{k_{ry}\cot\theta_i(r_\mathrm{p}+r_\mathrm{s})}{k_0} & r_s\end{bmatrix}\begin{bmatrix}\widetilde{E}_i^{\mathrm{H}}\\ \widetilde{E}_i^{\mathrm{V}}\end{bmatrix}, \tag{S3}$$

$k_0 = 2\pi/\lambda$ is the wave number in a vacuum.

After passing through the sample, the preselection state is corrected as [3]:

$$|\psi_i\rangle=\cos(\pm\alpha)\ |\mathrm{H}\rangle+\sin(\pm\alpha)\ |\mathrm{V}\rangle, \tag{S4}$$

where the incident light is horizontally (H) polarized. Here Figure 2(b) indicates that OR angle provided by chirality could rotate the polarization plane of incident light and $\pm\alpha$ represents the opposite direction of OR angle provided by opposite chirality. Then, when the reflected light beam passes through the second Glan polarizer (P2), which is orthogonal to P1:

$$|\psi_f\rangle=|\mathrm{V}\rangle, \tag{S5}$$

Then, the weak value amplification and propagation amplification of observed outcome in weak measurement system is given by [4].

$$A_w=\frac{\langle\psi_f|\widetilde{A}|\psi_i\rangle}{\langle\psi_f|\psi_i\rangle}, \tag{S6a}$$

$$A_w^{mod}=F|A_w|. \tag{S6b}$$

According to the light intensity distribution of reflect light beam from Eqs. S1-S5 and theoretical calculation formula abs^2[j], the theoretical result of light spot splitting when post-selected state is fixed in V polarization can be obtained as:

$$j[x,y]=\frac{1}{2\pi}\int_{-\infty}^{+\infty}\int_{-\infty}^{+\infty}\tilde{E}_i\left(k_{ix}+k_{iy}\right)\left(r_s\sin\alpha-\frac{k_{iy}}{k_0}\left(r_p+r_s\right)\cos\alpha\cot\theta_i\right)\ \exp[\mathrm{I}\left(k_{ix}x+k_{iy}y\right)-\mathrm{I}z\frac{k_{ix}^2+k_{iy}^2}{2k_0}], \tag{S7}$$

where x and y denote the unit orthogonal vector, the propagation distance z=250mm.

Finally, the final amplified transverse shift and light spot splitting of photonic SHE can be expressed [5, 6]:

$$\delta_y=\frac{z\, r_s(r_p+r_s)\cot\theta_i\sin 2\alpha}{(r_p+r_s)^2\cot^2\theta_i\cos^2\alpha+k_0^2w_0^2r_s^2\sin^2\theta_i}, \tag{S8}$$

$$j[x,y]=\frac{\sqrt{\frac{2}{\pi}}k_0w_0\left(-2\mathrm{I}y\left(r_p+r_s\right)\cos\alpha\cot\theta_i+r_s\left(k_0w_0^2+2\mathrm{I}z\right)\sin\alpha\right)\exp\left[-\frac{k_0(x+y)}{k_0w_0^2+2\mathrm{I}z}\right]}{(k_0w_0^2+2\mathrm{I}z)^2}. \tag{S9}$$

All the above theoretical results about amplified transverse shift and light intensity distribution of light spot are calculated on the mathematical calculation tool Wolfram Mathematica.

Figure S3 shows the spot splitting of $FAPbBr_3$ NCs with particle sizes form 5nm to 20nm, which can illustrate $FAPbBr_3$ NCs itself provides no OR angle. And Figure S4 reveals the spot splitting of rac-NEA and rac-NEA-coated $FAPbBr_3$ NCs, respectively. Here the corresponding OR angle is 0 and spot splitting keeps complete symmetry, which indicates rac provides no OR angle.

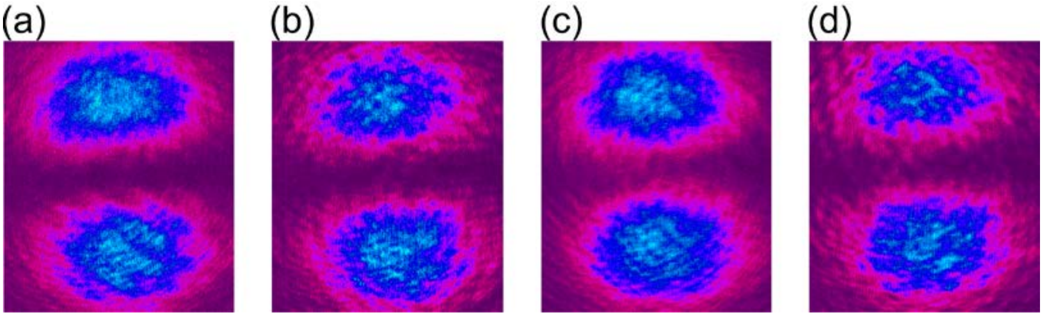

Figure S3. (a)-(d) Light spot splitting of non-NEA-coated $FAPbBr_3$ NCs with particle sizes form 5nm to 20nm.

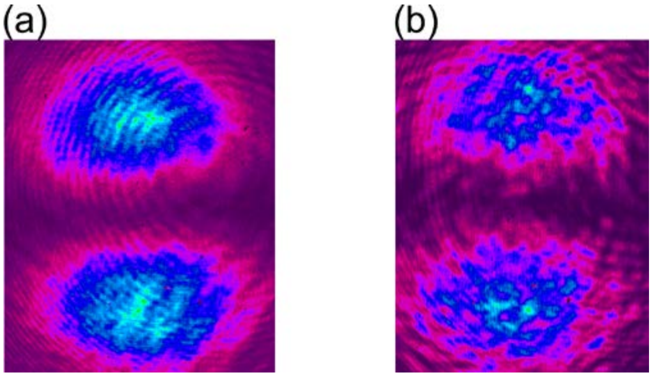

Figure S4. Light spot splitting of (a) rac-NEA ligands and (b) rac-NEA-coated $FAPbBr_3$ NCs.

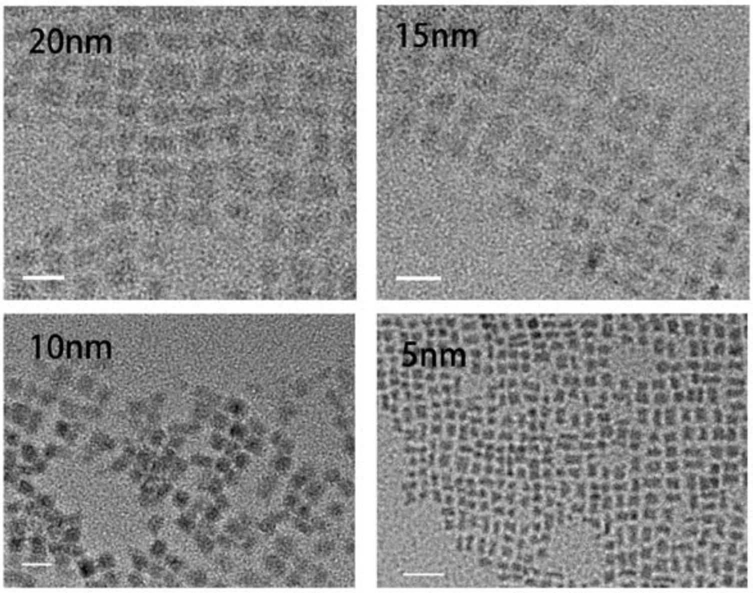

Figure S5. HR-TEM images of $FAPbBr_3$ NCs with different particle sizes, and scale bar are 20nm.

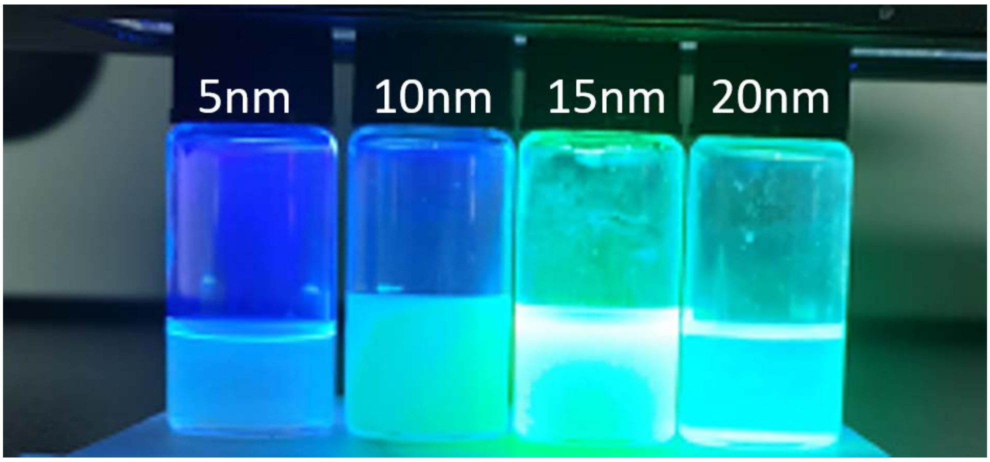

Figure S6. Luminescence of $FAPbBr_3$ NCs with different particle sizes under UV lamp.

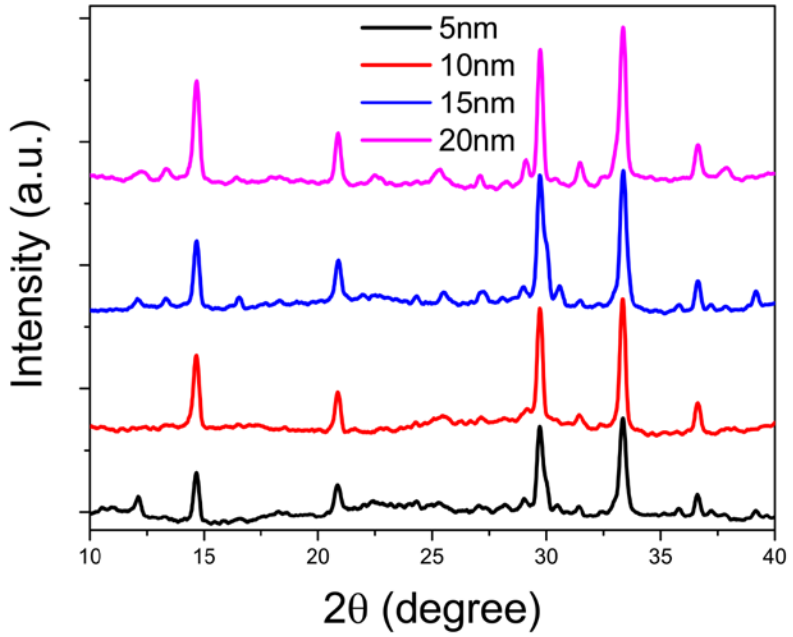

Figure S7. XRD of $FAPbBr_3$ NCs with different particle sizes

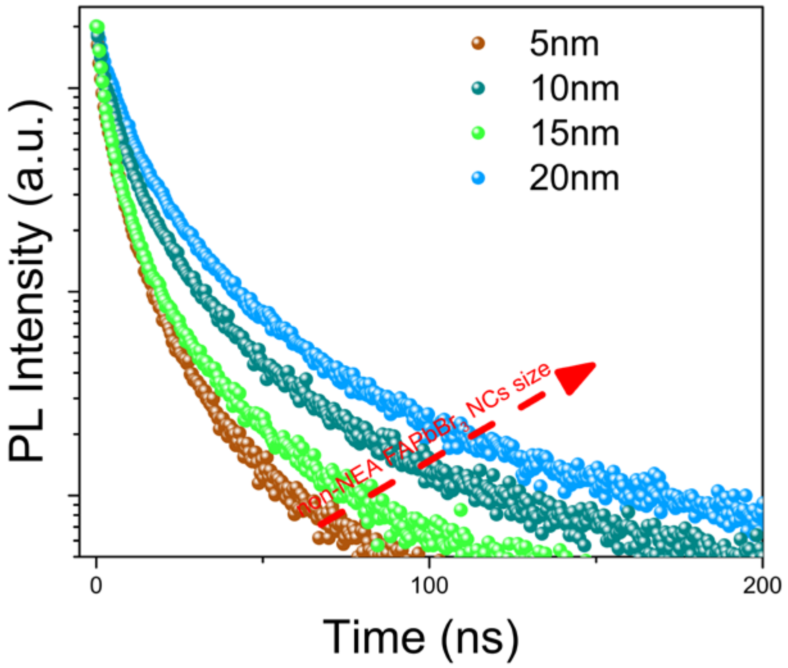

Figure S8. Time-resolved photoluminescence spectra of non-NEA-coated $FAPbBr_3$ NCs with four particle sizes.

Non-NEA-coated $FAPbBr_3$ NCs with different particle sizes were fitted with a single exponential in TRPL, and the results showed that the lifetime of non-NEA-coated $FAPbBr_3$ NCs increased from 4.22 ns to 10.41 ns with increase of particle size.

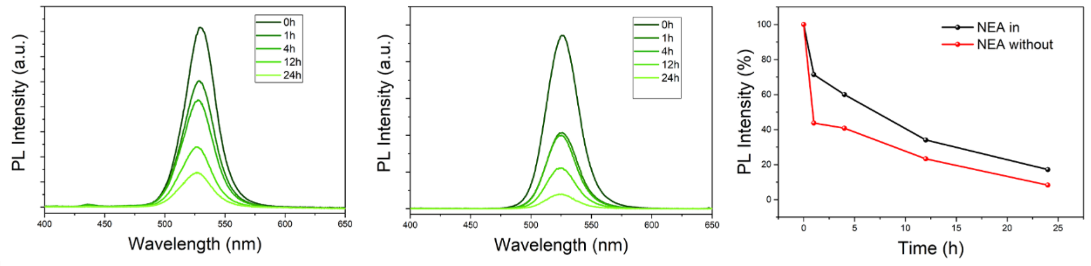

Figure S9. PL spectra of 20nm (a) NEA-coated $FAPbBr_3$ NCs and (b) non-NEA-coated $FAPbBr_3$ NCs over time, (c) PL intensity change.

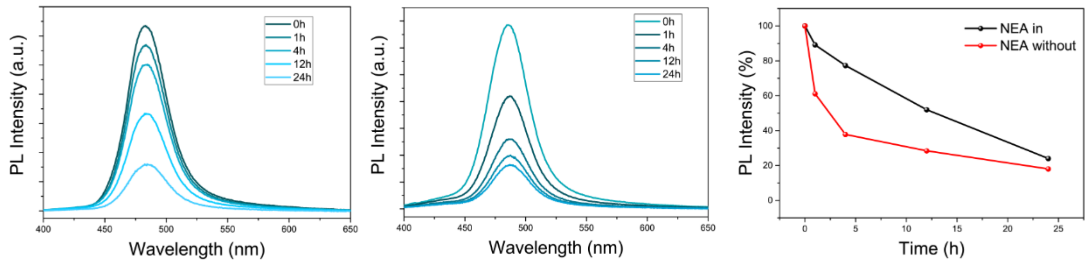

Figure S10. PL spectra of 15nm (a) NEA-coated $FAPbBr_3$ NCs and (b) non-NEA-coated $FAPbBr_3$ NCs over time, (c) PL intensity change.

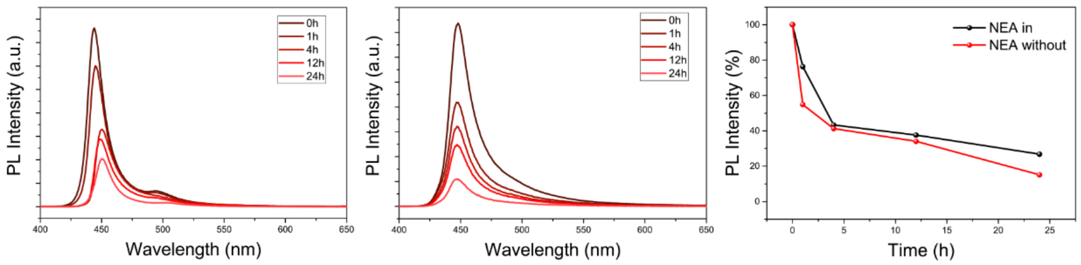

Figure S11. PL spectra of 10nm (a) NEA-coated $FAPbBr_3$ NCs and (b) non-NEA-coated $FAPbBr_3$ NCs over time, (c) PL intensity change

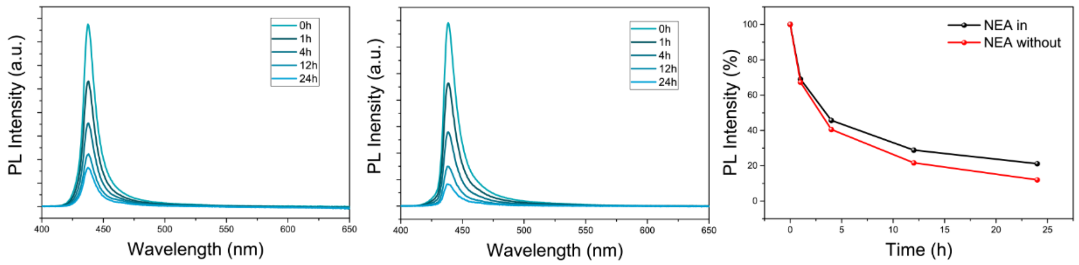

Figure S12. PL spectra of 5nm (a) NEA-coated $FAPbBr_3$ NCs and (b) non-NEA-coated $FAPbBr_3$ NCs over time, (c) PL intensity change.

Reference

[1] H. Luo, X. Zhou, W. Shu, S. Wen, and D. Fan, “Enhanced and switchable spin Hall effect of light near the Brewster angle on reflection,” Phys. Rev. A. 84(4),043806 (2011).

[2] M. Kim, D. Lee, Y. Kim, and J. Rho, “Generalized analytic formula for spin Hall effect of light: shift enhancement and interface independence,” Nanophotonics, https://doi.org/10.1515/nanoph-2021-0794 (2022).

[3] L. Xu, L. Lan, H. Wu, Z. Luo, Z. Zhang, H. Shi, T. Chang, P. Wu, C. Du, and H. Cui, “Measurement of chiral molecular parameters based on a combination of surface plasmon resonance and weak value amplification,” ACS sensors 5(8), 2398-2407 (2020).

[4] J. M. Ménard, A. E. Mattacchione, M. Betz, and H. M. van Driel, “Imaging the spin Hall effect of light inside semiconductors via absorption,” Opt. Lett. 34(15), 2312–2314 (2009).

[5] A. Aiello, N. Lindlein, C. Marquardt, and G. Leuchs, “Transverse Angular Momentum and Geometric Spin Hall Effect of Light,” Phys. Rev. Lett. 103(10), 100401–100401 (2009).

[6] K. Y. Bliokh, M. A. Alonso, E. A. Ostrovskaya, and A. Aiello, “Angular momenta and spin-orbit interaction of nonparaxial light in free space,” Phys. Rev. A. 82(6), 063825 (2010).

[7] Buck, U.; Meyer, H., SCATTERING ANALYSIS OF AR-CLUSTER BEAMS. Surf. Sci. 156 (JUN), 275-281 (1985).

[8] Das, T. K. Ilaiyaraja, P. Sudakar, C. Whispering Gallery Mode Enabled Efficiency Enhancement: Defect and Size Controlled CdSe Quantum Dot Sensitized Whisperonic Solar Cells. Scientific Reports. 8 (2018).

[9] Huang, W. J. Sun, R.; Tao, J. Menard, L. D. Nuzzo, R. G. Zuo, J. M., Coordination-dependent surface atomic contraction in nanocrystals revealed by coherent diffraction. Nature Materials 7 (4), 308-313 (2008).
